# Supplementary material for: A scoping review of interventions to improve strength training participation
Source: PLoS One. 2022 Feb 3;17(2):e0263218. doi: 10.1371/journal.pone.0263218 (PMC8812857; doi:10.1371/journal.pone.0263218)
Supplement: S2 File — (DOCX) [file pone.0263218.s002.docx]

***Sample Search Strategy***

**Search strategy from EMBASE**

| 1. strength training.mp. |
| --- |
| 2. exp resistance training/ |
| 3. resistance training.mp. |
| 4. muscle strengthening.mp. |
| 5. community program.mp. or exp community program/ |
| 6. intervention study.mp. or exp intervention study/ |
| 7. physical education.mp. or exp physical education/ |
| 8. promotion.mp. |
| 9. exp curriculum/ or curriculum.mp. |
| 10. initiative.mp. |
| 11. behaviour change.mp. or exp behavior change/ |
| 12. strategy.mp. |
| 13. 1 or 2 or 3 or 4 |
| 14. 5 or 6 or 7 or 8 or 9 or 10 or 11 or 12 |
| 15. 13 and 14 |
|  |

**Note. Exp: explode MESH term**

**/:MESH term**

**mp: Keyword**
