# Supplementary material for: A scoping review of interventions to improve strength training participation
Source: PLoS One. 2022 Feb 3;17(2):e0263218. doi: 10.1371/journal.pone.0263218 (PMC8812857; doi:10.1371/journal.pone.0263218)
Supplement: S3 File — (PDF) [file pone.0263218.s003.pdf]

S3- Coding Manual

ProACTIVE Specific Coding Instructions
Social support practical: speocify if information related to referral/resources, but can also be providing advice/BCTs
Social support emotional: Encouragement, praise
Social support unspecified: Refer to friends, colleagues, family for general support
Identification of self as role model: Also include others as role models
Tailoring: Just look for exemplary situations
Planning to facilitate behaviour e.g. planning what gyms you'll use: Problem solving
Add Participant BCT: if BCT was initiated by participant
Coding same BCT multiple times: Only recode when a new concept and code at the beginning of a concept
Only code BCT if it actually occurred, not just discussed it
Coding scheme for (highlight in comment box):
• Additional BCT (green):
• Not a BCT (yellow):
• Unsure (blue):
• Participant BCT (purple):
· New agreed upon code (Green)
Break up social support practical→ referrals to people, places, programs etc.
Break up instructions on how to perform the behaviour→ Also specify if: Technique, FITT (any of frequency, intensity, ti
Problem solving: Only include if both identification of barrier and solution
Review goal: Code when goal setting was already established and are revisiting the goal
Behavioural practice/rehearsal: Advise to try a gym code as Behavioral practice/ rehearsal
Supervision: Feedback on behaviour or monitoring of the behaviour by others without feedback *If it's not clear form th
Generalisation of a target behavior: If performed in the gym setting over time and then asked to transition to home setti
Feedback on behaviour: Specify if technology e.g., FitBit, Computer
Goal setting: if they set that the participants were required to complete 30 minutes, 2 days/week for 6 months
If talking about safety: code as safety not health consequences
Behavioural practice: Can be supervised exercise
When coding table, reference which part of the table you are coding in [square brackets]. Also, do not recode if it is alrea
When coding control group BCTs if not apparent put in brackets (not counted) for control group BCTs that are also condu
For BCTs in control only add (control only)
Note: Goal setting (mutually discussed vs prescribed- which is better)
If only referencing the 'benefits of ST' code as
