# Supplementary material for: A scoping review of interventions to improve strength training participation
Source: PLoS One. 2022 Feb 3;17(2):e0263218. doi: 10.1371/journal.pone.0263218 (PMC8812857; doi:10.1371/journal.pone.0263218)
Supplement: S4 File — (DOCX) [file pone.0263218.s004.docx]

**List of Included Studies**

1. Arikawa YA, Schmitz K. Adherence to a Strength Training Intervention in Adult Women. *J Act Phys Health* 2011;**8**:111-18
2. Baker K, LaValley MP, Brown C, Felson DT, Ledingham A, Keysor JJ. Efficacy of Computer-Based Telephone Counseling on Long-Term Adherence to Strength Training in Elderly Patients With Knee Osteoarthritis: A Randomized Trial. Arthritis Care Res. 2020;72: 982–990. doi:10.1002/acr.23921
3. Baker KR, Nelson ME, Felson TD, *et al.* The efficacy of home based progressive strength training in older adults with knee. *J. Rheumatol.* 2001;**28**:1655-65.
4. Davy BM, Winett RA, Savla J,*et al.* Resist diabetes:A randomized clinical trial for resistance training maintenance in adults with prediabetes. *PLoS ONE* 2017;***12***. 10.1371/journal.pone.0172610
5. Falcon A. Use of a dvd-based strength training program by breast cancer survivors in the home setting. *Diss. Abstr. Int. Sect. B Sci. Eng.* 2016;**76**.
6. Fetherman DL, Hakim RM, Sanko JP. A pilot study of the application of the transtheoretical model during strength training in older women. *J Women Aging* 2011;**23**:58–76. doi:10.1080/08952841.2011.540487
7. Jette AM, Lachman M, Giorgetti MM, *et al.* Exercise It's Never Too Late: The Strong-for-Life Program. *Am J Public Health* 1999;**89**.
8. Jette AM, Rooks D, Lachman M, *et al.* Home-based resistance training: Predictors of participation and adherence. *Gerontologist* 1998;**38**:412–21. doi:10.1093/geront/38.4.412
9. Kamada M, Kitayuguchi J, Abe T, *et al.* Community-wide promotion of physical activity in middle-aged and older Japanese: A 3-year evaluation of a cluster randomized trial. *Int J Behav Nutr Phys Act* 2015;**12**.
10. Kamada M, Kitayuguchi J, Inoue S, *et al.* A community-wide campaign to promote physical activity in middle-aged and elderly people: A cluster randomized controlled trial. *Int J of* *Behav Nutr Phys Act* 2013;***10***.
11. Latimer-Cheung AE, Arbour-Nicitopoulos KP, Brawley LR, *et al.* Developing physical activity interventions for adults with spinal cord injury. Part 2: Motivational counseling and peer-mediated interventions for people intending to be active. *Rehabil Psychol* 2013;**58**:307–15. doi:10.1037/a0032816
12. Lubans DR, Plotnikoff RC, Jung M, *et al.* Testing mediator variables in a resistance training intervention for obese adults with type 2 diabetes. *Psychology and Health*. 2012;***27***:1388-1404.
13. Lubans DR, Mundey CM, Lubans NJ, *et al.* Pilot randomized controlled trial: Elastic-resistance-training and lifestyle-activity intervention for sedentary older adults. *J Aging Phys Act* 2013;**21**:20–32
14. Mailey EL, Gasper R, Dlugonski D, Besenyi GM. Promoting Strength Training Among Baby Boomers: Message Framing Effects on Motivation and Behavior. Int J Behav Med. 2020. doi:10.1007/s12529-020-09939-9
15. Marinik EL, Kelleher S, Savla J, *et al.* The Resist Diabetes trial : Rationale , design , and methods of a hybrid ef fi cacy / effectiveness intervention trial for resistance training maintenance to improve glucose homeostasis in older prediabetic adults. *Contemp Clin Trials* 2014;**37**:19–32.

doi:10.1016/j.cct.2013.11.006

1. Mikesky AE, Mazzuca SA, Brandt KD, *et al.* Effects of strength training on the incidence and progression of knee osteoarthritis. *Arthritis Care Res 2006*;***55***:690-99.
2. Mikesky AE, Topp R, Wigglesworth JK, *et al.* Efficacy of a home-based training program for older adults using elastic tubing. *Eur J Appl Physiol* *1994*;**69**:316-20.
3. Millen JA, Bray SR. Promoting self-efficacy and outcome expectations to enable adherence to resistance training after cardiac rehabilitation. *J Cardiovasc Nurs* 2009;**24**:316–27. doi:10.1097/JCN.0b013e3181a0d256
4. Mullane SL, Bocchicchio VB, Crespo NC. Community-based resistance training program for preadolescent children. 2017;**40**:183–91. doi:10.1097/FCH.0000000000000157
5. Osuka Y, Jung S, Kim T, *et al.* Does attending an exercise class with a spouse improve long-term exercise adherence among people aged 65 years and older: A 6-month prospective follow-up study. *BMC Geriatr.* 2017;***17***. 10.1186/s12877-017-0554-9
6. Ott CD, Lindsey AM, Waltman NL, *et al.* Facilitative strategies, psychological factors, and strength/weight training behaviors in breast cancer survivors who are at risk for osteoporosis. *Orthop Nurs* 2004;**23**:45–52. doi:10.1097/00006416-200401000-00013
7. Papadopoulos CP, Jager JM. The effect of an educational program on strength-training

adherence in older adults. *Educ Gerontol* 2016*;****4****:*342-51. 10.1080/03601277.2015.1121752

1. Plotnikoff RC, Eves N, Jung M, *et al.* Multicomponent, home-based resistance training for obese adults with type 2 diabetes: A randomized controlled trial. *Int J Obes* 2010;**34**:1733–41. doi:10.1038/ijo.2010.109
2. Schmitz KH, Hannan PJ, Stovitz SD, *et al.* Strength training and adiposity in premenopausal women : Strong , Healthy , and Empowered study 1 – 4. 2007;**86**:566–72.
3. Schwartz AL, Winters-Stone K. Effects of a 12-month randomized controlled trial of aerobic or resistance exercise during and following cancer treatment in women. *Phys Sportsmed* 2009;**37**:62–7. doi:10.3810/psm.2009.10.1730
4. Sigal, RJ, Kenny GP, Boulé NG, *et al*. Effects of aerobic training, resistance training, or both on glycemic control in type 2 diabetes a randomized trial*. Ann Intern Med* 2007;**147**:357-69.
5. Sparrow D, Gottlieb DJ, Demolles D, *et al.* Increases in muscle strength and balance using a resistance training program administered via a telecommunications system in older adults. *Journals Gerontol - Ser A Biol Sci Med Sci* 2011;**66 A**:1251–7. doi:10.1093/gerona/glr138
6. Teychenne M, Ball K, Salmon J, *et al.* Adoption and maintenance of gym-based strength training in the community setting in adults with excess weight or type 2 diabetes: a randomized controlled trial. *Int J Behav Nutr Phys Act* 2015;**12**:1–9. doi:10.1186/s12966-015-0266-5
7. Vanroy J, Seghers J, Van Uffelen J, Boen F. Can a framed intervention motivate older adults in assisted living facilities to exercise? BMC Geriatr. 2019;19: 1–11. doi:10.1186/s12877-019-1060-z
8. Williams DM, Dunsiger S, Davy BM, *et al.* Psychosocial mediators of a theory-based resistance training maintenance intervention for prediabetic adults. *Psychol Heal* 2016;**31**:1108–24.

doi:10.1080/08870446.2016.1179740

1. Wilson ML, Strayer TE, Davis R,  *et al.* Use of an integrated research-practice partnership to improve outcomes of a community-based strength-training program for older adults: Reach and effect of lifelong improvements through fitness together (LIFT). *Int J Environ Res Public Health* 2018;***15***. 10.3390/ijerph15020237
2. Winett RA, Davy BM, Savla J, *et al.* Theory-based approach for maintaining resistance training in older adults with prediabetes: adherence, barriers, self-regulation strategies, treatment fidelity, costs. *Translational Behavioral Medicine* 2015;***5***:149-159. 10.1007/s13142-015-0304-5
3. Winters-Stone KM, Dobek J, Bennett JA, *et al.* The effect of resistance training on muscle strength and physical function in older, postmenopausal breast cancer survivors: A randomized controlled trial. *J Cancer Surviv* 2012;**6**:189–99. doi:10.1007/s11764-011-0210-x
4. Winters-Stone KM, Dobek JD, Nail LN, *et al.* Strength training stops bone loss and builds muscle in postmenopausal breast cancer survivors: A randomized, controlled trial. *Breast Cancer Res. Treat.* 2011;**127**:447-456.
5. Winters-Stone KM, Lyons KS, Dobek J, *et al.* Benefits of partnered strength training for prostate cancer survivors and spouses: results from a randomized controlled trial of the Exercising Together project. *J Cancer Surviv* 2016;***10***:633-44.
