# Supplementary material for: A scoping review of interventions to improve strength training participation
Source: PLoS One. 2022 Feb 3;17(2):e0263218. doi: 10.1371/journal.pone.0263218 (PMC8812857; doi:10.1371/journal.pone.0263218)
Supplement: Attachment — Submitted filename: PLoS ONE review comments.docx [file pone.0263218.s005.docx]

**Review comments to authors:**

The current study is on a topic of relevance and the idea is innovative. This scoping review has salience towards the planning of behaviour change interventions for strength training.

Of greater importance will be the systematic review regarding the efficacy of these interventions which is currently being under-taken (as indicated by the authors in the Discussion section). One option is to combine both papers or submit as companion papers. There is probably enough information focus to warrant both papers.

The methodological approach for this scoping review appears to have some limitations. It is not clear how the authors selected the search terms; more detail is warranted. Why were other relevant databases not selected (e.g. SPORTDiscus, Cinahl, Scopus)? We believe that some key studies have not been included in this review. It was not entirely clear how the authors selected the included studies, i.e. clarification is required regarding the selection criteria for study selection. On page 8 – it is reported that ‘any study design; was included in the search strategy. However this paper reports on ‘intervention studies’.

Overall, the manuscript requires major revisions including more focused writing and structuring within each section. Specific examples and suggestions are detailed below.

***Introduction***

Overall, the introduction needs to be written in more focused and clear language. For example, the first sentence should be edited to “*International guidelines recommend that adults and older adults should engage in muscle strengthening activities at least twice weekly*.” The sentence starting on line 98 also needs editing, it should read “*ST is especially important for older adults, where increased strength helps support continued function and independence”.*

Instead of using the term “*ST behaviour change interventions*”, which reads quite awkwardly. It is recommended that authors reword this to “*ST interventions that use/include/utilizes behaviour change strategies*”.

It is also recommended that when authors discuss the low rates of meeting the ST guidelines, that they compare these to the prevalence of meeting the aerobic guidelines.

On a number of locations, the authors mention ‘strengthening activities’, e.g. line 106. It is recommended that the authors reword this to ‘muscle strengthening’ or ‘muscle strengthening activities’ and use this throughout the manuscript.

The sentence starting on line 110 is awkward and requires rewording. For example, “*When compared to aerobic activities (e.g. walking), the perceived knowledge and effort required to effectively perform muscle strengthening activities (e.g. specific techniques, cost of equipment, knowledge of how much ST to perform/ how to progress)* *can be deterring.”* Authors also need to reference this sentence.

The sentence starts on line 113 is awkward and needs rewording. The authors need to be really clear what they are referring to i.e. interventions targeting a particular type of exercise. For example, “*Given the unique barriers to ST, it may be that interventions targeting ST require different behaviour change strategies than those targeting aerobic exercise or ST combined with other physical activities.”*

The last paragraph of the introduction is awkward and repetitive in places, please revise.

***Methods***

The method needs to be written in more focused language and be more structured. For example:

It is recommended that authors use an appropriate subheading for the first paragraph.

Page 6. This paragraph is confusing and wordy, please revise using more focused language.

Page 6. The last sentence of the paragraph should be removed.

It is also recommended that authors remove the ‘Stage 1’, ‘Stage 2’ etc from each of the subheadings as it is expected that each section follows in a chronological fashion.

Page 8. In the study selection. While the authors include the inclusion criteria and exclusion criteria. Authors also state on line 191 “*Several strength training interventions have been conducted to date, the inclusion of articles with a measure of ST participation as an outcome was selected to help distinguish behavioural interventions from general ST training interventions*”. Please clarify this.

On page 9. Authors are using the heading “Charting the data”, please change to heading “Data extraction” as this terminology is used in the abstract. It is important to be consistent with the language used throughout the manuscript.

On page 9, line 215, the authors have written 2.4.1 – please remove.

On page 9, change the subheading “*Collating, summarizing, and reporting the results*” to “*Analysis*”

***Results***

On page 10, under “Study characteristics” and “Intervention characteristics”, instead of spelling out all the information that is already presented in Table 1, do a brief summary. Alternatively, remove Table 1 and Table 2 and keep the text.

Please include the range of participants i.e. ranging between X to 3500.

It is also recommended that the authors edit Table 3. In the first column, add in the reference number as opposed to stating the author and year. In Table 3, the authors mention 1-RM in the ‘Exercise volume’ column and the number of sets in the exercise intensity? 1-RM is a measure of intensity, please go through the table and amend accordingly. In addition, second column under ‘Exercise frequency’, this information is often repeated under the ‘Intervention procedure’ column. Please revise and avoid repeating information.

***Discussion***

Overall, the discussion requires major revisions, including being written in a more structured and focused language. Some examples listed below:

Page 20, line 302, avoid ‘I’ or ‘We’ statements, please amend to “*The present review identified 24 unique interventions for improving ST behaviour*”.

In the second paragraph “Opportunity to test theory” the authors are suggesting a number of theories to include, HAPA, SDT and TPB, however the authors do not justify why these ones should be tested? Have they been used in other aerobic and/or aerobic + RT interventions? The authors should remove the sentence on line 321-324 or reword as it doesn’t really fit into the context. All those constructs are part of SCG.

The ‘Intervention characteristics: consideration of practical interventions” paragraph is confusing and hard to follow. The authors needs to rethink their discussion points.

Throughout the discussion, the authors could remove the first sentence under each subheading or remove the subheadings, as the first sentence is repetition from the previous paragraph.

Page 24, line 407, please remove the statement “frequency does not infer effectiveness” unless the authors can reference this. Also reword the next sentence to make it more generalised i.e. other BCT’s may be effective (equally, less or more), but we don’t know as these haven’t been tested.

**References**

Some references are listed two or more times eg Lubans ,et al 2013. Please go through the reference list and remove duplicates where applicable.
