# Supplementary material for: A scoping review of interventions to improve strength training participation
Source: PLoS One. 2022 Feb 3;17(2):e0263218. doi: 10.1371/journal.pone.0263218 (PMC8812857; doi:10.1371/journal.pone.0263218)
Supplement: Attachment — Submitted filename: Response to Reviewers- PLOS ONE June 2021.docx [file pone.0263218.s007.docx]

**Response to Reviewers**

We appreciate the reviewers thoughtful and valuable comments which have greatly helped to strengthen our manuscript. We have responded in detail to all their comments below.

**Legend:**

Reviewer Comments: Black text

Response to Reviewers: Blue italicized text

*Note: Line numbers are referenced for the track changes copy

PONE-D-20-27748
A scoping review of strength training behaviour change interventions: Future research and practical applications

PONE-D-20-27748R1
A scoping review of interventions to improve strength training participation
PLOS ONE

Dear Dr. Ma,

Thank you for submitting your manuscript to PLOS ONE. After careful consideration, we feel that it has merit but does not fully meet PLOS ONE’s publication criteria as it currently stands. Therefore, we invite you to submit a revised version of the manuscript that addresses the points raised during the review process.

Thank you for addressing the reviewers' concerns. There are a few remaining minor style edits required before acceptance. Please see below for specific comments.

Please submit your revised manuscript by Aug 07 2021 11:59PM. If you will need more time than this to complete your revisions, please reply to this message or contact the journal office at plosone@plos.org. When you're ready to submit your revision, log on to https://www.editorialmanager.com/pone/ and select the 'Submissions Needing Revision' folder to locate your manuscript file.

Please include the following items when submitting your revised manuscript:

- A rebuttal letter that responds to each point raised by the academic editor and reviewer(s). You should upload this letter as a separate file labeled 'Response to Reviewers'.
- A marked-up copy of your manuscript that highlights changes made to the original version. You should upload this as a separate file labeled 'Revised Manuscript with Track Changes'.
- An unmarked version of your revised paper without tracked changes. You should upload this as a separate file labeled 'Manuscript'.

If you would like to make changes to your financial disclosure, please include your updated statement in your cover letter. Guidelines for resubmitting your figure files are available below the reviewer comments at the end of this letter.

If applicable, we recommend that you deposit your laboratory protocols in protocols.io to enhance the reproducibility of your results. Protocols.io assigns your protocol its own identifier (DOI) so that it can be cited independently in the future. For instructions see: <http://journals.plos.org/plosone/s/submission-guidelines#loc-laboratory-protocols>. Additionally, PLOS ONE offers an option for publishing peer-reviewed Lab Protocol articles, which describe protocols hosted on protocols.io. Read more information on sharing protocols at <https://plos.org/protocols?utm_medium=editorial-email&utm_source=authorletters&utm_campaign=protocols>.

We look forward to receiving your revised manuscript.

Kind regards,

Stephanie Prince Ware, PhD
Academic Editor
PLOS ONE

*RESPONSE: We sincerely thank the reviewers and PLOS One for their time and thought in review. We have outlined our revisions point-by-point as indicated by the text in blue.*

Journal Requirements:

Please review your reference list to ensure that it is complete and correct. If you have cited papers that have been retracted, please include the rationale for doing so in the manuscript text, or remove these references and replace them with relevant current references. Any changes to the reference list should be mentioned in the rebuttal letter that accompanies your revised manuscript. If you need to cite a retracted article, indicate the article’s retracted status in the References list and also include a citation and full reference for the retraction notice.
*RESPONSE: The reference list has been reviewed.*

Additional Editor Comments (if provided):

1. In the abstract, please include the search date ranges.

*RESPONSE: Added “(inception-December 2020)”*

2. In the abstract, PsycINFO is misspelled.

*RESPONSE: Corrected, apologies for the oversight.*

3. In the abstract, please describe the type of synthesis used.

*RESPONSE: Line 40: Have included “Data were synthesized using descriptive and frequency reporting.”*

4. Page 5, there are two "Methods" and "Methodology" headings, please keep just the "Methods" heading.

*RESPONSE: Removed the “Methodology” heading.*

5. Page 6, line 128: the research question is not needed as it is essentially a rephrasing of the objectives provided in the background.

*RESPONSE: The research question has been removed.*

6. Page 7, line 141: PsycINFO is misspelled.

*RESPONSE: Corrected, apologies for the oversight.*

7. Page 7: Can the authors confirm this is the complete search strategy? Please provide at least 1 full strategy for a sample database in supplemental material.

*RESPONSE: Yes, we can confirm that the full search strategy is included in supplemental material.*

8. Page 10, line 215: please spell out 5 (i.e. five).

*RESPONSE: Corrected.*

9. Page 10, line 220: please spell out 2 (i.e. two).

*RESPONSE: Corrected.*

10. Table 1, please include age ranges for the populations.

*RESPONSE: Age ranges have been added.*

11. Page 12, line 237: please spell out 7 (i.e. seven).

*RESPONSE: Corrected.*

[Note: HTML markup is below. Please do not edit.]

Reviewers' comments:

Reviewer's Responses to Questions

**Comments to the Author**

1. If the authors have adequately addressed your comments raised in a previous round of review and you feel that this manuscript is now acceptable for publication, you may indicate that here to bypass the “Comments to the Author” section, enter your conflict of interest statement in the “Confidential to Editor” section, and submit your "Accept" recommendation.

Reviewer #1: All comments have been addressed

Reviewer #2: All comments have been addressed

2. Is the manuscript technically sound, and do the data support the conclusions?

The manuscript must describe a technically sound piece of scientific research with data that supports the conclusions. Experiments must have been conducted rigorously, with appropriate controls, replication, and sample sizes. The conclusions must be drawn appropriately based on the data presented.

Reviewer #1: Yes

Reviewer #2: Yes

3. Has the statistical analysis been performed appropriately and rigorously?

Reviewer #1: N/A

Reviewer #2: Yes

4. Have the authors made all data underlying the findings in their manuscript fully available?

The [PLOS Data policy](http://www.plosone.org/static/policies.action#sharing) requires authors to make all data underlying the findings described in their manuscript fully available without restriction, with rare exception (please refer to the Data Availability Statement in the manuscript PDF file). The data should be provided as part of the manuscript or its supporting information, or deposited to a public repository. For example, in addition to summary statistics, the data points behind means, medians and variance measures should be available. If there are restrictions on publicly sharing data—e.g. participant privacy or use of data from a third party—those must be specified.

Reviewer #1: Yes

Reviewer #2: Yes

5. Is the manuscript presented in an intelligible fashion and written in standard English?

PLOS ONE does not copyedit accepted manuscripts, so the language in submitted articles must be clear, correct, and unambiguous. Any typographical or grammatical errors should be corrected at revision, so please note any specific errors here.

Reviewer #1: No

Reviewer #2: Yes

6. Review Comments to the Author

Please use the space provided to explain your answers to the questions above. You may also include additional comments for the author, including concerns about dual publication, research ethics, or publication ethics. (Please upload your review as an attachment if it exceeds 20,000 characters)

Reviewer #1: The authors have adequately addressed the comments.

However, there are a number of places throughout the paper where the grammar, structure and wording could be improved. I suggest some minor editing to this effect.

*RESPONSE: We thank the reviewer for their suggestions in this and the previous revision. To address issues related to grammar, structure, and wording, we had a professional editor review and make such changes. Changes are highlighted using track changes throughout.*

Reviewer #2: (No Response)

7. PLOS authors have the option to publish the peer review history of their article ([what does this mean?](https://journals.plos.org/plosone/s/editorial-and-peer-review-process#loc-peer-review-history)). If published, this will include your full peer review and any attached files.


If you choose “no”, your identity will remain anonymous but your review may still be made public.


**Do you want your identity to be public for this peer review?** For information about this choice, including consent withdrawal, please see our [Privacy Policy](https://www.plos.org/privacy-policy).

Reviewer #1: No

Reviewer #2: **Yes:** Jason Bennie

[NOTE: If reviewer comments were submitted as an attachment file, they will be attached to this email and accessible via the submission site. Please log into your account, locate the manuscript record, and check for the action link "View Attachments". If this link does not appear, there are no attachment files.]

While revising your submission, please upload your figure files to the Preflight Analysis and Conversion Engine (PACE) digital diagnostic tool, <https://pacev2.apexcovantage.com/>. PACE helps ensure that figures meet PLOS requirements. To use PACE, you must first register as a user. Registration is free. Then, login and navigate to the UPLOAD tab, where you will find detailed instructions on how to use the tool. If you encounter any issues or have any questions when using PACE, please email PLOS at [figures@plos.org](mailto:figures@plos.org). Please note that Supporting Information files do not need this step.

*Confirming that our Figures have been uploaded and modified using the suggested PACE tool.*
