# Supplementary material for: A scoping review of interventions to improve strength training participation
Source: PLoS One. 2022 Feb 3;17(2):e0263218. doi: 10.1371/journal.pone.0263218 (PMC8812857; doi:10.1371/journal.pone.0263218)
Supplement: Attachment — Submitted filename: Response to Reviewers- PLOS ONE Jan 2022.docx [file pone.0263218.s008.docx]

**Response to Reviewers**

PONE-D-20-27748
A scoping review of strength training behaviour change interventions: Future research and practical applications

We appreciate the reviewers’ thoughtful and valuable comments throughout the review process. Their feedback has greatly helped to strengthen our manuscript. We have responded in detail to the final comment from the editor below.

**Legend:**

Reviewer Comments: Black text

Response to Reviewers: Blue italicized text

*Note: Line numbers are referenced for the track changes copy

Reviewer: You cite a PROSPERO protocol (CRD42019120251) but PROSPERO does not register scoping reviews. The project was originally registered as a standard systematic review, therefore please add to your paper an explicit mention of this and explain why the study began as a standard systematic review and then was converted to a scoping review.

*Response: LINE 423-431: “Second, this review was originally registered with PROSPERO (CRD42019120251) as a systematic review with two research questions. Question one (“what are the effects of strength training participation interventions?”) will be addressed in a separate systematic review. Question two in the registered protocol was defined as “what BCTs, theories, and modes of delivery are used in strength training interventions currently?’. Given the nascency and heterogeneity of the literature, we changed to a scoping review methodology for this specific question. Specifically, it was more appropriate to map the current state of the literature and suggest a research agenda that addresses current gaps than to assess the effectiveness of these intervention components, therefore warranting a scoping review methodology.”*

PONE-D-20-27748R2

A scoping review of interventions to improve strength training participation

PLOS ONE

Dear Dr. Ma,

Thank you for submitting your manuscript to PLOS ONE. After careful consideration, we feel that it has merit but does not fully meet PLOS ONE’s publication criteria as it currently stands. Therefore, we invite you to submit a revised version of the manuscript that addresses the point raised below.

You cite a PROSPERO protocol (CRD42019120251) but PROSPERO does not register scoping reviews. The project was originally registered as a standard systematic review, therefore please add to your paper an explicit mention of this and explain why the study began as a standard systematic review and then was converted to a scoping review.
